# Medical Education and Practice at Early Juntendo During the First Three Generations of Directors: Analysis from the Standpoint of History of Medicine

**DOI:** 10.14789/ejmj.JMJ26-0014-OA

**Published:** 2026-05-20

**Authors:** TATSUO SAKAI

**Affiliations:** 1 Department of History of Medicine, Juntendo University Faculty of Medicine, Tokyo, Japan; 1 Department of History of Medicine, Juntendo University Faculty of Medicine, Japan

**Keywords:** early Juntendo, medical education, traditional Western medicine, modern medicine, surgery

## Abstract

**Objectives:**

This study aimed to clarify the characteristics of medical education and clinical practice at early Juntendo under its first three generations of directors.

**Materials:**

We collected and analyzed historical documents related to early Juntendo, as well as bibliographic materials on medical and surgical textbooks published in the early modern and modern periods.

**Methods:**

The textbooks in early Juntendo were specified and contents of their originals were analyzed and evaluated.

**Results:**

The first three directors of Juntendo―Sato Taizen, Sato Takanaka, and Sato Susumu―studied Western medicine and surgery in Nagasaki or abroad and subsequently introduced and developed these practices at Juntendo in three successive stages. In the first stage (1838-1862), following Taizen’s return from Nagasaki, Taizen and Takanaka translated pre-modern surgical textbooks, particularly those by Chelius, and applied their contents in clinical practice. In the second stage (1862-1875), after Takanaka’s training under Pompe van Meerdervoort, he translated semi-modern textbooks by Stromeyer and Wunderlich and established systematic medical education. In the third stage (1875-1921), after Susumu’s return from Germany, modern surgery incorporating anesthesia and antiseptic techniques was introduced, and Takanaka translated Niemeyer’s modern textbook of internal medicine. These developments reflected the rapid modernization of Western medicine in the nineteenth century, particularly in surgery and internal medicine.

**Conclusions:**

The first three directors of Juntendo continuously adopted the most advanced phases of Western medicine and surgery of their time, thereby establishing Juntendo as a leading medical school and hospital in modern Japan.

## Introduction

Juntendo University is the oldest Western-style medical school in Japan, having been established in 1838 as a Dutch medical school, Wada-juku, in Edo by its first director, Sato Taizen. Under the leadership of its second director, Sato Takanaka — one of the most prominent physicians and medical scholars of his time — Juntendo developed into a major center for modern medical education in Sakura. Takanaka later served as president of Daigaku Toko, the predecessor of the Faculty of Medicine at the University of Tokyo, and subsequently founded Juntendo Hospital in Yushima. The third director, Sato Susumu, introduced advanced surgical techniques from Europe and transformed Juntendo Hospital into one of the most respected medical institutions in Tokyo. The early history of Juntendo has been comprehensively documented in Juntendo-shi (“History of Juntendo”)^1)^. During the first three generations of directors (hereafter referred to as “early Juntendo”), the institution played a pivotal role in the transplantation and development of Western medicine in Japan during the late Edo and early Meiji periods, together with medical education led by German physicians at the University of Tokyo^2)^.

Recent scholarship has demonstrated that Western medicine in the early modern period (sixteenth to eighteenth centuries) differed fundamentally from modern medicine established in the nineteenth century and thereafter^3)^. In early modern Europe, university-trained physicians competed for patients with empirically trained surgeons and apothecaries, and hospitals functioned primarily as charitable institutions rather than as centers of systematic medical treatment^4)^. Medical knowledge taught at universities was largely based on classical Galenic doctrines and later mechanistic theories, although it gradually incorporated findings from anatomical dissection and bedside observation^5)^. The nineteenth century marked a major transitional period from traditional to modern Western medicine. This transformation was characterized by the development of pathological anatomy, the emergence of objective diagnostic methods, and the establishment of evidence-based therapeutic strategies. Surgery and internal medicine, in particular, underwent rapid modernization through the introduction of anesthesia, antiseptic techniques, and new disease concepts. The historical development of Juntendo in the nineteenth century closely paralleled this broader transformation of Western medicine. However, the specific processes through which medical knowledge, textbooks, and clinical practices were introduced, adapted, and institutionalized at Juntendo have not yet been fully clarified from a comparative historical perspective.

In this study, we therefore aimed to elucidate the process of medical modernization at early Juntendo by analyzing the evolution of medical education and clinical practice under its first three generations of directors. By examining the textbooks, teaching materials, and clinical activities employed at each stage, this study seeks to situate Juntendo’s development within the broader context of nineteenth- century Western medical history.

## Materials and Methods

This study was conducted through a four-step historical and bibliographic investigation of medical education and practice at early Juntendo and of related Western medical literature.

In the first step, teaching materials and textbooks used at early Juntendo were identified and extracted from Juntendo-shi^1)^ and from online bibliographic databases, including CiNii Books^6)^.

In the second step, original editions of these teaching materials and textbooks, as well as information on their authors, were examined through direct analysis of books held at the Department of History of Medicine, Juntendo University, and through online resources, including the National Diet Library Digital Collections^7)^.

In the third step, the publication histories and dissemination of the original Western medical textbooks were investigated using the online catalogs of major international libraries, including the National Library of Medicine (NLM) Catalog^8)^, Bayerische Staatsbibliothek (BSB) Discover^9)^, and the National Library of the Netherlands (KB) Catalog^10)^.

In the fourth step, original textbooks were obtained either in print or in digital format from online repositories, including the Internet Archive^11)^ and Google Books^12)^. Their contents were systematically analyzed with respect to structure, theoretical framework, and clinical orientation.

Through these procedures, the study evaluated the characteristics of medical education and clinical practice at each developmental stage of early Juntendo in relation to contemporary trends in Western medicine. The study was a historical literature-based investigation and therefore required no ethical approval.

## Results

### Historical development of early Juntendo

Juntendo University has the longest tradition among medical universities in Japan, and its early period under the first three generations of directors occupies a particularly important position in the history of Japanese medicine. The institution was founded in 1838, when Sato Taizen established Wada-juku in Edo and introduced Dutch medicine. After relocating to Sakura in 1843, Taizen opened Juntendo and developed an advanced and highly regarded surgical practice. The second director, Sato Takanaka, studied modern medicine under Pompe van Meerdervoort in Nagasaki from 1860 to 1862 and significantly enhanced Juntendo’s reputation through systematic medical education. After serving as president of Daigaku Toko, he established Juntendo Hospital in Yushima, Tokyo. The third director, Sato Susumu, studied modern surgery at the University of Berlin from 1869 to 1875. Upon his return, he introduced advanced surgical techniques and transformed Juntendo into a highly sophisticated and popular hospital in Tokyo (Table 1).

**Table 1:** Time table of the first three generations of directors at Juntendo

**First Director: Sato Taizen (1804-1872/5/16)** 1804: born in Kitamikata (now: Kanagawa prefecture), father: Sato Tosa (public representative), mother: Fuji. 1830-35: studied Dutch medicine under Adachi Choshun. 1835-38: studied Dutch language in Nagasaki from disciples of Siebold. 1838: opened Wada-juku in Edo and taught Dutch medicine. 1843: moved to Sakura, opened Juntendo and taught and practiced surgery. 1853: became han’i (“doctor of feudal domain”) of Sakura-han. 1859: retired from Juntendo. 1862: left Sakura and lived in Yokohama. 1872/5/16: died in Tokyo. **Second Director: Sato Takanaka (1827/5/3-1882/7/23)** 1827/5/3: born in Komigawa (now: Chiba prefecture), father: Yamaguchi Hosen (physician, Komigawa han’i), mother: En. 1839-42: studied medicine under Ando Buntaku. 1842: entered Wada-juku and learned Dutch medicine. 1843: moved to Sakura with Sato Taizen 1853: adopted by Sato Taizen. 1859: became director of Juntendo. 1860-62: studied modern medicine in Nagasaki from Pompe van Meerdervoort. 1862-68: taught modern medicine at Juntendo in Sakura. 1869-73: appointed to the Daihakase (“great doctor”, president) at Daigaku Toko (“East college of university”, predecessor of University of Tokyo Faculty of Medicine) 1873-75: opened Juntendo at Shitaya-Neribei-cho in Tokyo. 1875: opened Juntendo hospital at Yushima in Tokyo. 1883/7/23: died in Tokyo. **Third Director: Sato Susumu (1845/12/23-1921/7/25)** 1845/12/23: born in Ohta (now; Ibaragi prefecture), father: Takawa Seibei (sake brewer), mother: Tami. 1859: entered Juntendo to study Dutch medicine. 1866-67: married with Shizu (Takanaka’s daughter) and was adopted by Takanaka. 1868/6-12: participated as the chief military doctor in the Boshin War to treat wounded soldiers. 1869/6-75/7: studied abroad at the Berlin University in Germany and absorbed modern surgery with anesthesia and antiseptic techniques. 1875/7: taught and practiced surgery at Juntendo hospital. 1877: army surgeon general during the Seinan Rebellion. 1878/12-1882/10: director of the Rikugun Hon Byoin (“Army Main Hospital”). 1883/7: became director of Juntendo. 1895/5-8: army surgeon general during the First Sino-Japanese War and treated wounded Li Hongzhang. 1904/5-05/12: army surgeon general during the Russo-Japanese War. 1921/7/25: died in Tokyo.

### Teaching materials and textbooks in early Juntendo

The development of Rangaku (Dutch learning) in Japan was strongly influenced by the publication of Kaitai Shinsho (“New Text on Anatomy”, 1774) and Ihan Teiko (“Concise Model of Medicine”, 1805). When Sato Taizen began studying Dutch medicine in 1830, these works had already laid the foundation for Western medical learning. The development of medical education at early Juntendo was closely associated with three major “breakthrough” events: Taizen’s training in Nagasaki (1835-1838), Takanaka’s study under Pompe van Meerdervoort (1860-1862), and Susumu’s training in Germany (1869-1875). Based on these events, the history of early Juntendo can be divided into three major stages, preceded by a preparatory period (Table 2).

**Table 2:** History of medical education and practice at early Juntendo and the breakthrough events in it

<Breakthrough event 0> Rangaku (Dutch learning) studies after Kaitai Shinsho (1774), Ihan Teiko (1805) and other translations. **[Pre-history] Sato Taizen studied Dutch medicine under Adachi Choshun (1830-38).** Taizen studied Dutch medicine from Japanese translations. < Breakthrough event 1> Taizen studied Dutch language in Nagasaki from disciples of Siebold (1835-38) **[1st stage] Sato Taizen opened Wada-Juku in Edo (1838-43). Sato Taizen and Takanaka taught and practiced medicine in Juntendo at Sakura (1843-62)** Translation and absorption of Dutch medicine. Practice of surgical operations. < Breakthrough event 2> Sato Takanaka studied modern medicine in Nagasaki from Pompe van Meerdervoort (1860-62) **[2nd stage] Sato Takanaka taught and practiced medicine at Juntendo in Sakura and moved to Tokyo (1862-75)** Lecture of basic and clinical medicine Practice of medicine at Juntendo in Sakura Appointment of Sato Takanaka to the daihakase at Daigaku Toko (1869-73) Opening of Juntendo hospital in Neribei-cho (1873) and moving to Yushima (1875) < Breakthrough event 3> Sato Susumu studied abroad in Germany (1869/6-75/7) and absorbed modern surgery with anesthesia and antiseptic techniques. **[3rd stage] Sato Susumu as the director taught and practiced surgery at Juntendo hospital together with Sato Takanaka. (1875-1921).** Lecture and practice of medicine at Juntendo hospital.

During the first stage, Taizen, Takanaka, and their colleagues focused primarily on translating Dutch medical texts for educational purposes. The original sources included Dutch translations of German works, such as Most’s medical encyclopedia and Chelius’s handbooks of surgery and ophthalmology. These texts formed the foundation of early medical education and surgical practice at Juntendo (Table 3).

**Table 3:** The translated medical textbooks and their originals at early Juntendo in the first stage (1838-62)

Sato Taizen: Toka Shusei (“Pox clinic collection”) [Hufeland, CW: Bemerkungen über die natürlichen und inoculirten Blattern. (1798) German original, Dutch translation] Sato Takanaka: On-shi Geka-sejutsu Taizen (“Mr. On’s surgical operations collection”) [Onsenoort, AGv: De operative heelkunde stelselmatig voorgedragen. (1822-36) Dutch original] Hayashi Doukai: Tsuzoku Waatoru Yakushouron (“Popular Water’s pharmacy theory”, 1840 self-preface) [Water, JAvd: Beknopt doch zoo veel mogelijk volledig handboek voor de leer der geneesmiddelen : (materies medica). (1829) Dutch original] Sato Taizen: Ganka Hatsuun (“Ophthalmology elucidation”, 1843); Sato Taizen: Most Gyuto-hen (“Most cow pox book”, 1849); Sato Takanaka: Mo-shi Korera-setsu (“Mr. Mo’s cholera explanation”, 1858) [Most, GF: Encyklopädie der gesammten medicinischen und chirurgischen Praxis. (1833-34) German original, Dutch translation] Sato Taizen: Sekkotsu Biyo (“Osteopathy memorandam”); Sato Takanaka: Yougaku Zensho (“Surgery complete book”) [Chelius, MJ: Handbuch der Chirurgie zum Gebrauche bei seinen Vorlesungen. (1822-23) German original, Dutch translation] Sato Takanaka: Se-shi Ganka-sho (“Mr. Se’s ophthalmology book”) [Chelius, MJ: Handbuch der Augenheilkunde (1839-43) German original, Dutch translation]

In the second stage, Takanaka introduced systematic instruction in basic and clinical medicine, drawing on lecture notes from Pompe van Meerdervoort and Dutch translations of contemporary European textbooks. Major sources included works by Wunderlich, Stromeyer, and Hyrtl. This period marked a transition toward more structured and comprehensive medical education. (Table 4)

**Table 4:** The translated medical textbooks of early Juntendo in the second stage (1862-75)

Sato Takanaka: Unteruri Kakketsurouryou-hen Zen (“Wunderlich hemoptysis tuberculosis book all”, 1861) [Wunderlich, CRA: Handbuch der Pathologie und Therapie. (1846-1854) German original, Dutch translation] Sato Takanaka: Geka Ihou (“Surgical therapy method”, 1865) [Stromeyer, GFL: Handbuch der Chirurgie. Band 1. (1844) German original, Dutch translation] Sato Takanaka: Sutoromeru Houi-ron (“Stromeyer gunshot wound theory”, 1865) [Stromeyer, GFL: Maximen der Kriegsheilkunst. (1861) German original, Dutch translation] Sato Takanaka: Pompe Kaibo-sho (“Pompe’s anatomy book”) [Pompe van Meerdervoort’s anatomy lecture note at Nagasaki] Anon.: Sutokuharudoto Shamitsu-sho (“Stöckhardt’s chemistry book”) [Stöckhardt, JA: Die Schule der Chemie, oder erster Unterricht in der Chemie (1846) German original, Dutch translation] Anon.: Hirutoru Kaibo-sho (“Hyrtl’s anatomy book”) [Hyrtl, J: Lehrbuch der Anatomie des Menschen (1846) German original, Dutch translation] Anon.: Unterurii Naika-sho (“Wunderlich’s internal medicine book”) [Wunderlich, CRA: Grundriss der speciellen Pathologie und Therapie (1858) German original, Dutch translation]

During the third stage, under the leadership of Susumu, modern surgery incorporating anesthesia and antiseptic techniques was firmly established at Juntendo Hospital. Susumu published original surgical textbooks based on contemporary German literature, particularly Billroth’s works, while Takanaka translated Niemeyer’s textbook of internal medicine. This stage represented the full institutionalization of modern Western medicine at Juntendo. (Table 5)

**Table 5:** The translated medical textbooks of early Juntendo in the third stage (1875-1921)

Anon.: Juntendo Iji-zasshi (“Juntendo medical journal”, vol. 1-8, 1875-77) Sato Susumu: Geka Tsuron (“Surgery, general discussions”, 25 vols, 1876-80) [Billroth, T: Die allgemeine chirurgische Pathologie und Therapie in fünfzig Vorlesungen. Ein Handbuch für Studirende und Aertzte. (1863) German original] Sato Susumu: Geka Kakuron (“Surgery, specific discussions”, 18 vols, 1880-86) [Linhart, W: Compendium der chirurgischen Operationslehre. 4th ed. (1874) German original] [Bardeleben, A: Lehrbuch der Chirurgie und Operationslehre : besonders für das Bedürfnis der Studirenden. 7th ed. (1874-76) German original] [König, F: Lehrbuch der speciellen Chirurgie : für Aerzte und Studirende. (1875-1877) German original] [Pitha, Fv: Krankheiten der Extremitäten. (1868) German original] [Billroth, T: Chirurgische Klinik, Wien, 1868 [1869-1876] Erfahrungen aus dem Gebiete der praktischen Chirurgie. (1870-79) German original] Sato Takanaka: Saishuroku (“Salvation documents”, 14 vol, 1879)) [Niemeyer, F: Lehrbuch der speciellen Pathologie und Therapie (1858-61) German original, Dutch translation]

### Surgery textbooks as employed in the early Juntendo

Surgical practice underwent dramatic development in the nineteenth century. WTG Morton’s public demonstration of ether anesthesia in 1846 and J Lister’s introduction of antiseptic methods greatly improved surgical safety. At Juntendo, different surgery textbooks were adopted at different stages: Chelius’s work (1822-23) in the first stage, Stromeyer’s (1844) in the second, and Sato Susumu’s (1876-86) in the third.

**Figure 1:**
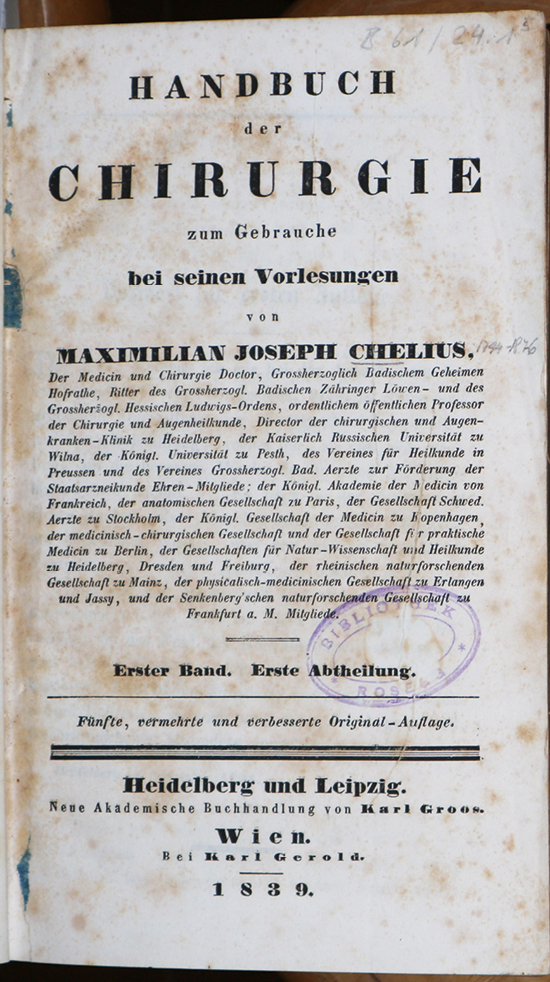
Chelius MJ “Handbuch der Chirurgie” 5th edition (1839-40), volume one, title page. Reproduced from Chelius MJ. Handbuch der Chirurgie, zum Gebrauche bei seinen Vorlesungen. Fünfte, vermehrte und verbesserte Original-Auflage. Heidelberg und Leipzig: Karl Groos, 1839-41. The original publication is in the public domain. Photograph taken by Tatsuo Sakai (personal collection).

Chelius, Maximilian Joseph (1794-1876) was professor of surgery at Heidelberg. His surgery textbook (1822-1823)^13)^ reflected pre-modern concepts, emphasizing traditional pathological classifications and focusing mainly on limb amputations for military injuries. The book contained eight parts: parts 1-7 on surgical pathology dealing (1) inflammation, (2) disruption of connection, (3) coherence, (4) foreign bodies, (5) neoplasm, (6) loss of parts, and (7) excess of parts, and part 8 on surgical operation. The surgical pathology was archaic in style, since it contained the ancient concepts of “disruption of connection” (part 2) and “contra-natural” (part 2-3, part 3) and stereotyped classification of degeneration (part 5), loss (part 6) and excess (part 7) of body parts succeeded from the ancient Galen’s pathology. The inflammation was regarded as a category of illness as in Sauvage’s nosology system, instead of a kind of pathological process as in the modern medicine. The individual surgical operations dealt mostly amputation of limbs which was necessary to treat military wounds. (Table 6) (Figure 1)

**Table 6:** Chelius, MJ: Handbuch der Chirurgie zum Gebrauche bei seinen Vorlesungen. (1822-23) contents in English

Part 1. Inflammation. I. Inflammation in general. II. Specific types of inflammation: (1) erysipelas, (2) burns, (3) frostbite, (4) boils, (5) carbuncles. III. Inflammation in certain organs: (1) tonsils, (2) parotid gland, (3) breasts, (4) urethra, (5) testicle, (6) lumbar muscles, (7) nails, (8) joints. Part 2. Disruption of physical connection. Part 2-1. Disruption of the connection: (1) fresh (wounds, fractures), (2) old (ulcers, fistulas). Part 2-2. Altered position of parts: (1) dislocations, (2) herniation, (3) prolapse, (4) curvatures. Part 2-3. Contra-natural expansion: (1) arterial aneurism, (2) venous varicose. Part 3. Contra-natural coherence. Part 4. Presence of foreign bodies: (1) from outside, (2) abnormal accumulation, (3) pathological products, (4) stony concretions. Part 5. Degeneration of body parts or neoplasm: (1) enlarged tongue, (2) goiter, (3) enlarged clitoris and labia, (4) warts, (5) calluses, (6) horny neoplasms, (7) bony neoplasms, (8) neoplasms of dura mater, (9) fatty tumors, (10) follicle-tumors, (11) cartilaginous bodies in the joints, (12) fleshy tumors, (13) medullary tumors, (14) polyps, (15) cancers. Part 6. Loss of body parts: (1) organic replacement, (2) mechanical replacement, (3) artificial hands, (4) artificial upper arm, (5) artificial nose and ears, (6) replacement of hard palate. Part 7. Excess of body parts: (1) supernumerary fingers and toes, (2) supernumerary teeth, (3) double nose. Part 8. Surgical operations. Part 8-1. Elementary procedures of surgical operations. Part 8-2. Surgical operations general discussions. Part 8-3. Amputations in the continuity of individual limbs. Part 8-4. Amputations in the joints.

Stromeyer, Georg Friedrich Louis (1804-1876) was professor of surgery at Erlange, München, Freiburg and Kiel. His surgery textbook (1844)^14)^ represented a transitional stage. While its structure was modern, much of its content remained traditional, and visceral surgery was largely excluded due to limited anesthetic and antiseptic techniques. The book contained both general and specific discussions as in the usual modern medical textbooks. The general discussion dealing with surgical pathology was traditional, including hyperemia, inflammation, dyscrasia hypertrophic and parasitic formations, functional nervous diseases and mechanical injuries. The specific discussion dealing with surgical therapy was classical, including skin and cellular tissue (i.e. loose connective tissue), lymphatic vessels and glands, veins, arteries, nerves and locomotor organs. (Table 7)

**Table 7:** Stromeyer, GFL: Handbuch der Chirurgie. Band 1. (1844) contents in English

Introduction I. Theoretical surgery general discussion 1. Hyperemia 2. Inflammation: (1) results, (2) results in detail, (3) treatment, (4) suppuration, (5) induration, (6) softening, (7) gangrene, (8) risk of gangrene, (9) ulcer 3. Dyscrasias: (1) pyemia, (2) scrofula, (3) eczema, (4) gout, (5) scurvy 4. Hypertrophic and parasitic formations: (1) medullary spondylitis, (2) cancerous tumors, (3) sebaceous cyst, (4) lipoma, (5) sarcoma, (6) follicle tumors, (7) hydatids, (8) cartilaginous tumors 5. Functional nervous diseases or neuroses: (1) erethic neuroses, (2) neuralgia, (3) alcoholism, (4) delirium nervosum, (5) convulsions, (6) tetanus, (7) rheumatism, (8) paralysis 6. Mechanical injuries: (1) concussions and contusions, (2) wounds II. Theoretical surgery special discussion 1. Diseases of the skin and cellular tissue: (1) erythema, (2) erysipelas 2. Diseases of the lymphatic vessels and lymphatic glands 3. Diseases of the veins: (1) phlebitis, (2) venous dilatation and varicose 4. Diseases of the arteries: (1) arteritis, (2) aneurysms 5. Organic diseases of the nerves: (1) neuritis, (2) nerve contusions, (3) nerve tumors, neuromas 6. Diseases of the locomotor organs: (1) bone inflammation, (2) joint inflammation, (3) bone hypertrophies and bone tumors, (4) inflammation of individual joints, (5) injuries of joints, (6) fractures

**Figure 2:**
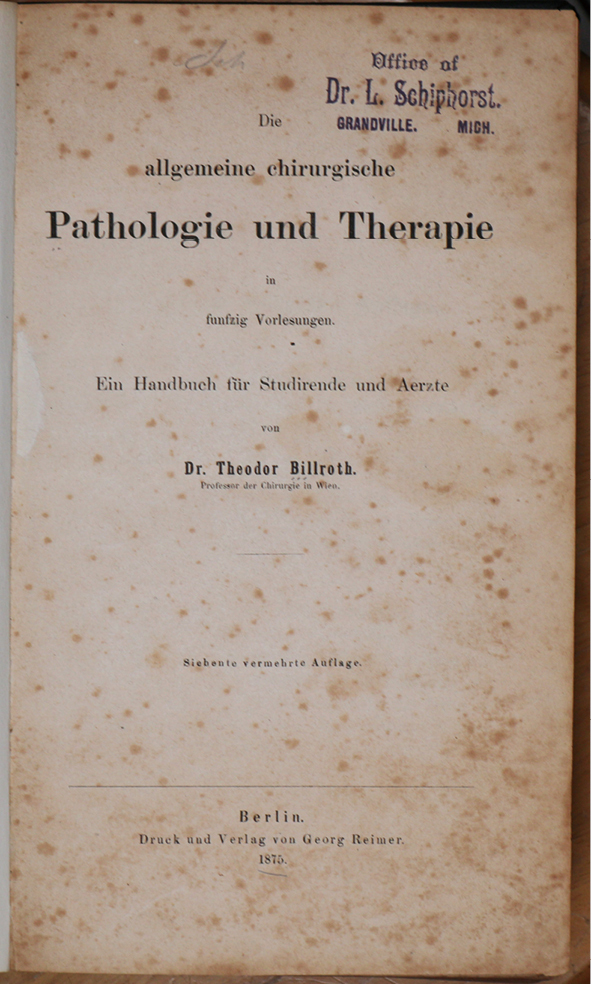
Billroth T “Die allgemeine chirurgische Pathologie und Therapie” 7th edition (1875), title page. Reproduced from Billroth T. Die allgemeine chirurgische Pathologie und Therapie in fünfzig Vorlesungen. Ein Handbuch für Studirende und Aertzte. Siebente vermehrte Auflage. Berlin: Georg Reimer, 1875. The original publication is in the public domain. Photograph taken by Tatsuo Sakai (personal collection).

Sato Susumu, the third director of Juntendo, studied abroad at the Berlin university and absorbed modern surgery with anesthesia and disinfection maneuvers. His lecture at Juntendo hospital was published as “Juntendo Iji-zasshi” (“Juntendo medical journal”, vol. 1-8, 1875-77). His surgery textbooks “Geka Tsuron” (“Surgery, general discussions”, 25 vols, 1876-80, revised ed., 1882) was written accordingly to Billroth’s surgical pathology “Die allgemeine chirurgische Pathologie und Therapie in fünfzig Vorlesungen” (1863), and “Geka Kakuron” (“Surgery, specific discussions”, 18 vols, 1880-86, revised ed., 1903) was written with reference to several surgical textbooks including Linhart’s “Compendium der chirurgischen Operationslehre” 4th ed. (1874), Bardeleben’s “Lehrbuch der Chirurgie und Operationslehre” 7th ed. (1874-76), König’s “Lehrbuch der speciellen Chirurgie” (1875-77), Pitha’s “Krankheiten der Extremitäten” (1868) and Billroth’s “Chirurgische Klinik, Wien, 1868 [1869-1876] Erfahrungen aus dem Gebiete der praktischen Chirurgie” (1870-79). (Figure 2)

Sato Susumu’s textbooks fully incorporated modern surgical principles, including anesthesia and disinfection. They addressed both general pathology and advanced operative techniques, including visceral surgery. His surgery general discussion textbook (1882)^15)^ contained 20 parts mainly on surgical pathology mainly on trauma and pathological process of locomotive organs. His surgery specific discussion textbook (1903-07)^16)^ contained 16 parts mainly of surgical operation for local diseases arranged from head to foot including diseases of cranial, thoracic, abdominal and pelvic visecra. (Table 8, Table 9)

**Table 8:** Sato Susumu: Geka Tsuron (“Surgery, general discussions”, 1882) contents in English

1. Simple lacerations of the soft tissues 2. Stab wounds 3. Contusion 4. Contusions and lacerations of the soft tissues 5. Simple fractures 6. Open fractures and osteomyelitis 7. Injuries of the joints 8. Gunshot wounds 9. Burns and frostbite 10. Acute inflammations of the soft tissues caused by trauma 11. Acute inflammations of the bones, periosteum and joints 12. Gangrene 13. Complications of wounds and inflammations 14. Chronic inflammations of the soft tissues 15. Ulcers 16. Chronic periostitis and osteoporosis 17. Chronic arthritis 18. Deformation of joint caused by congenital and acquired diseases 19. Venous dilatation and aneurysms 20. Tumor diseases.

**Table 9:** Sato Susumu: Geka Kakuron (“Surgery, specific discussions”, 1903) contents in English

1. Cranial trauma 2. Cranial diseases 3. Facial surgical diseases 4. Nasal and sinus diseases 5. Lip diseases 6. Jaw bones diseases 7. Oral and pharyngeal diseases 8. Ear diseases 9. Cervical diseases 10. Thoracic diseases 11. Trauma and diseases of abdomen 12. Urogenital diseases 1) Kidney diseases 2) Bladder diseases 3) Urethra diseases 4) Penis diseases 5) Scrotum diseases 6) Capsules, testis, epididymis diseases, together with male genital diseases 7) Seminal vesicle diseases 8) Prostate diseases 9) Cowper’s gland diseases 10) Sensory disorders of male genital organs 13. Vertebral column injuries and diseases 14. Upper limb surgery 15. Pelvis surgery 16. Lower limb surgery

### Medicine textbooks in early Juntendo

In the nineteenth century, pathological anatomy transformed medical concepts of disease. Modern medicine came to regard diseases as entities with specific causes, such as organ dysfunction (diseases of individual organs), pathogens (infection), or cellular abnormalities (neoplasm). In contrast, traditional Western medicine focused primarily on symptoms, representing abnormal bodily condition such as headache, cardiac palpitation, diarrhea and so on. Thus, in the modern medicine the sickness was treated by curative therapy to remove the cause of disease, whereas in the Western traditional medicine the sickness was treated by symptomatic therapy to alleviate physical and mental suffering. At Juntendo, medical education reflected this transition. No formal medical textbooks were used in the first stage. Wunderlich’s works (1858) were adopted in the second stage, and Niemeyer’s (1858-61) in the third.

**Figure 3:**
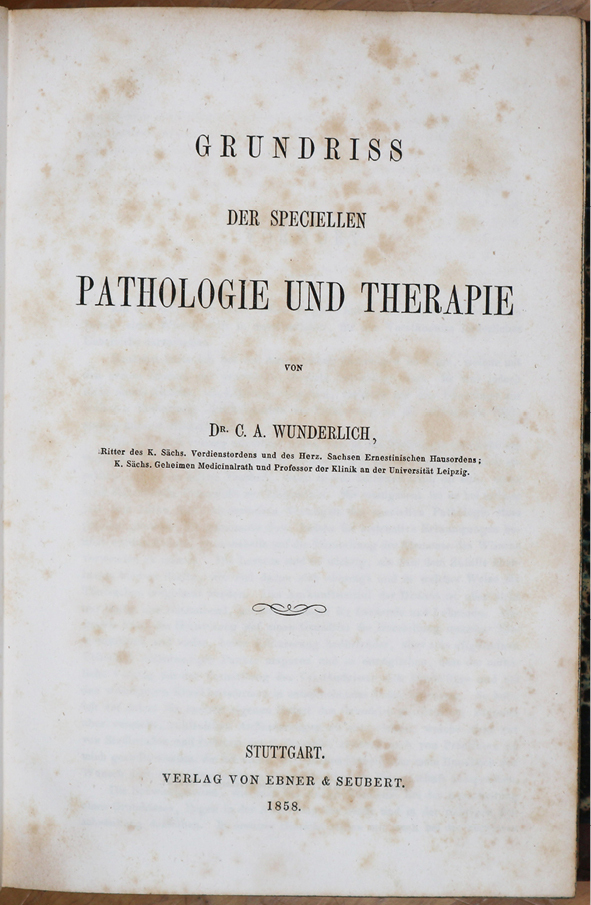
Wunderlich CRA “Grundriss der speciellen Pathologie und Therapie” (1858), title page. Reproduced from Wunderlich CRA. Grundriss der speciellen Pathologie und Therapie. Stuttgart: Ebner & Seubert, 1858. The original publication is in the public domain. Photograph taken by Tatsuo Sakai (personal collection).

Wunderlich, Carl Reinhold August (1815-1877) was professor of medicine at Tübingen and Leipzig. His medicine textbook (1858)^17)^ represented an eclectic and semi-modern approach, combining traditional pathological theories with emerging organ-based concepts. The book was divided into four parts. (A) [Introduction] containing seven chapters dealt with general discussion of pathology. (B) [Disease forms of tissue] containing 12 chapters dealt with pathological processes postulated to occur in the individual tissues. (C) [Local pathology] containing nine chapters dealt with diseases of individual organs. (D) [Specific constitutional diseases and affections with multiple localizations] containing two chapters dealt with systemic diseases, intoxication and infection. The textbooks contained in part the modern elements represented by diseases of individual organs in (C), but also the traditional elements such as ideological pathology in (A) and speculative disease category of the tissues in (B). (Table 10) (Figure 3)

**Table 10:** Wunderlich, CRA: Grundriss der speciellen Pathologie und Therapie (1858) contents in English

**[Introduction]** I. The causes and origins of disorders II. Disorders due to their spatial and temporal retention III. Basic forms of local disorders IV. Basic forms of systemic disorders V. Consequences of illness VI. Symptoms and classification of diseases VII. Therapy **[Disease forms of tissue]** I. Diseases of cellular or connective tissue II. Diseases of the serous membranes III. Diseases of the mucous membranes IV. Diseases of the skin and its appendages V. Affections of fibrous tissues VI. Affections of muscles VII. Affections of bones VIII. Affections of cartilage IX. Disorders of the ligaments of bones X. Affections of vessels XI. Diseases of the nervous system XII. Diseases of the secretory and vascular glands **[Local pathology]** I. Affections of the nerve centers and their adjacent sheaths II. Anomalies of the skull and vertebral column III. Affections of the scalp and facial regions IV. Affections of the respiratory organs V. Affections of the central circulatory organs VI. Diseases of the digestive tract and its appendages VII. Diseases of the urogenital system VIII. Disorders of the pelvis, abdominal walls, mammary glands, and general teguments of the trunk IX. Affections of the extremities **[Specific constitutional diseases and affections with multiple localizations]** I. Autogenic constitutional diseases II. Intoxications and infections

**Figure 4:**
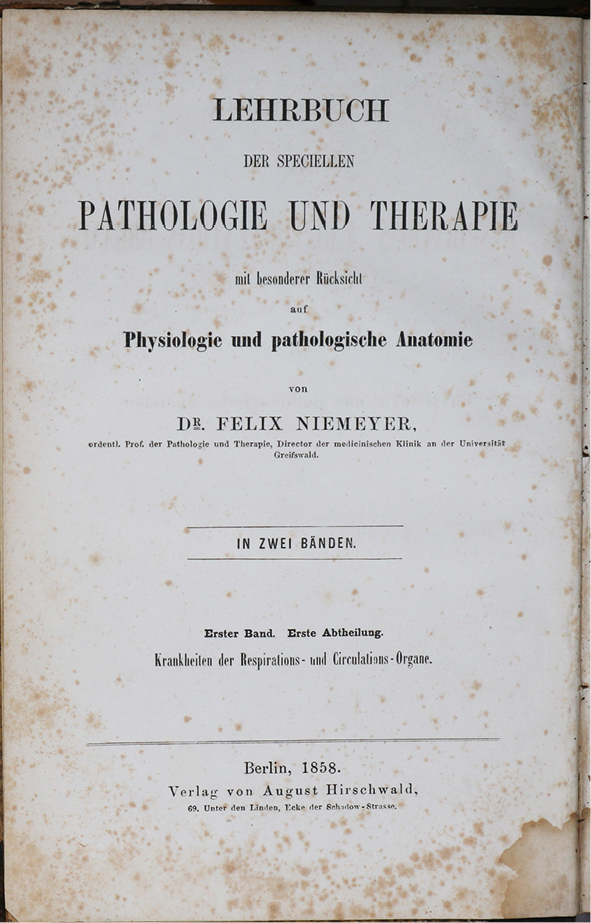
Niemeyer F “Lehrbuch der speciellen Pathologie und Therapie” (1858-61), volume one, title page. Reproduced from Niemeyer F. Lehrbuch der speciellen Pathologie und Therapie, mit besonderer Rücksicht auf Physiologie und pathologische Anatomie. Berlin: August Hirschwald, 1858-61. The original publication is in the public domain. Photograph taken by Tatsuo Sakai (personal collection).

Niemeyer, Felix (1820-1871) was professor of inner medicine at Greifswald. His medicine textbook (1858-61)^18)^, by contrast, reflected a fully modern approach. Diseases were systematically classified according to organ systems, and traditional speculative theories were excluded. The book was divided into two volumes and four parts and contained ten groups of diseases (nine local and one systemic). The nine groups of local diseases were arranged according to the functional organ systems; (1) respiratory organs, (2) circulatory organs, (3) digestive organs, (4) liver and biliary tract, (5) urinary organs, (6) reproductive organs, (7) nervous system, (8) skin, and (9) locomotor organs. The one group of systemic diseases concerned infectious diseases and nutritional abnormalities. The textbook contained no traditional elements of ideological pathology or speculative disease categories. (Table 11) (Figure 4)

**Table 11:** Niemeyer, F: Lehrbuch der speciellen Pathologie und Therapie (1858-61) contents in English

**Volume 1, part 1:** Diseases of the respiratory and circulatory organs with special consideration of physiology and pathological anatomy Diseases of the respiratory organs: 4 sections ([1] larynx, [2] trachea and bronchi, [3] lung parenchyma, [4] pleura) Diseases of the circulatory organs: 3 sections ([1] heart, [2] pericardial sac, [3] large vessels) **Volume 1, part 2:** Diseases of the digestive organs, liver, and spleen with special consideration of physiology and pathological anatomy Diseases of the digestive organs: 6 sections ([1] oral cavity, [2] pharynx, [3] esophagus, [4] stomach, [5] intestines, [6] omentum) Diseases of the liver and biliary tract: 2 sections ([1] liver, [2] biliary tract) **Volume 2, part 1:** Diseases of the urinary and reproductive organs, nerve centers, and nerves with special consideration of physiology and pathological anatomy Diseases of the urinary organs: 4 sections ([1] kidney, [2] renal pelvis and ureter, [3] urinary bladder, [4] urethra) Diseases of the reproductive organs Diseases of the male reproductive organs: 1 section Diseases of the female reproductive organs: 3 sections ([1] ovary, [2] uterus, [3] vagina) Diseases of the nervous system: 4 sections ([1] brain, [2] spinal marrow and meninges, [3] peripheral nerves, [4] neurosis with unknown anatomical basis) **Volume 2, part 2:** Diseases of the skin, locomotor organs, and constitutional diseases with special consideration of physiology and pathological anatomy Diseases of the skin: 1 section Diseases of the locomotor organs: 1 section Constitutional diseases: 3 sections and appendix ([1] acute infectious diseases, [2] chronic infectious diseases, [appendix] zoonotic diseases, [4] general nutritional abnormalities)

## Discussions

The present study revealed that medical education and practice in early Juntendo developed through three stages, incorporating Western medicine as it evolved from early modern traditions (16th-18th centuries) to modern medicine (19th-21st centuries). In the first stage (1838-1862), early Juntendo translated and studied pre-modern surgical textbooks by Chelius (1822-23). In the second stage (1862-1875), semi-modern textbooks of surgery by Stromeyer (1844) and of medicine by Wunderlich (1858) were translated and examined. In the third stage (1875-1925), modern surgical textbooks by Sato Susumu (1876-80, 1880-86) were published, and Niemeyer’s modern medical textbook (1858-61) was translated. (Table 12)

**Table 12:** Medicine and surgery textbooks in the early Juntendo

**(1) First stage at Wada-juku in Edo and at Juntendo in Sakura (1838-1862)** [Surgery] Chelius, MJ “Handbuch der Chirurgie zum Gebrauche bei seinen Vorlesungen” (1822-23) Pre-modern; Ancient concept of disease, authoritative and formal contents. [Medicine] No textbooks of internal medicine. **(2) Second stage at Juntendo in Sakura (1862-1875)** [Surgery] Stromeyer, GFL “Handbuch der Chirurgie. Band 1” (1844) Semi-modern; General and specific discussions, non- anesthetic, unsterilized, trauma and superficial tumors. [Medicine] Wunderlich, CRA “Grundriss der speciellen Pathologie und Therapie” (1858) Semi-modern: Including (1) disorders of tissues, (2) disorders of organs, (3) constitutional disorders; Eclectic containing both modern elements (diseases of individual organs) and traditional elements (ideological pathology and speculative disease categories). **(3) Third stage at Juntendo Hospital in Tokyo (1875-1921)** [Surgery] Sato Susumu “Geka Tsuron” (“Surgery, general discussions”, 25 vols, 1876-80); Sato Susumu “Geka Kakuron” (“Surgery, specific discussions”, 18 vols, 1880-86) Modern; With anesthesia and disinfection maneuvers, diseases of head, neck, chest, abdomen and pelvis, and limbs. [Medicine] Niemeyer, F “Lehrbuch der speciellen Pathologie und Therapie” (1858-61) Modern; Including disorders of organs; Excluding traditional elements of ideological pathology and speculative disease categories.

Western traditional medicine in the early modern period differed substantially in structure and content from modern medicine. These differences were most clearly reflected in medical education. In modern medicine, curricula and university institutions integrated basic medicine, which investigates the human body and disease mechanisms, with clinical medicine, which focuses on patient care. By contrast, early modern Western medicine was organized around four principal subjects: (A) medical theory, (B) medical practice, (C) anatomy and surgery, and (D) botany, pharmacy, and chemistry^19)^. Medical theory (theoretica) emphasized theoretical foundations derived mainly from Galenic humoral theory and later mechanical theories. Textbooks were often published under the title “Institutiones medicae”, such as Leonhart Fuchs’s (1555)^20)^, Daniel Sennert’s (1611)^21)^ and Hermann Boerhaave’s (1708)^22)^. Medical practice (practica) focused on individual diseases, addressing diagnosis, treatment, and prognosis. These textbooks, commonly titled “Practica medicinae”, covered both localized diseases and systemic conditions such as fevers^23)^. In the late eighteenth and early nineteenth centuries, medical practice was gradually replaced by nosology, which aimed to classify diseases systematically^24)^. Anatomical education was revitalized by Vesalius’s Fabrica (1543), leading many medical schools to establish anatomical theaters for dissection^25)^. Botany was essential for identifying medicinal herbs, and classical texts such as Dioscorides’ Materia Medica remained influential, alongside newly compiled illustrated herbals^26, 27)^. (Table 13)

**Table 13:** Comparison and differences between the Western traditional medicine in the early modern (16th-18th century) and the modern medicine in the modern times (19th-21th century)

**[Western traditional medicine: 16th-18th c.]** *Medical education:* four subjects (A) Medical theory (B) Medical practice (C) Anatomy / surgery (D) Botany / pharmacy / chemistry *Surgery:* non-anesthetic, non-antiseptic Operation: risky, short time Indication: trauma, superficial tumors *Medicine:* medical practice, nosology Sickness: physical symptoms (ex: headache, dyspnea, diarrhea, fevers etc.) Subjective diagnosis: complaints, life history, signs (uroscopy, pulse taking) Symptomatic treatment: to relieve symptoms	**[Modern medicine: 19th-21th c.]** *Medical education:* basic and clinical Basic medicine: biomedical investigation ex: anatomy, physiology, pathology etc. Clinical medicine: treatment of diseases ex: internal medicine, surgery etc. *Surgery:* anesthetic, antiseptic Operation: secure, long-lasting Indication: diseases of visceral organs *Medicine:* internal medicine Diseases: disorder of organs, infection, cancer Objective diagnosis: clinical laboratory investigation, pathological anatomy Curative therapy: to remove cause of diseases

Modern surgery advanced far beyond early modern surgery due to major technological innovations in the mid-nineteenth century, particularly anesthesia (1846) and sterilization techniques (1860s). These developments enabled safer and more complex operations on internal organs. In contrast, early modern surgery focused mainly on trauma, such as fractures, dislocations, war injuries, and superficial tumors.

The transition from early modern to modern medicine involved three major transformations. First, concepts of disease changed fundamentally^28)^. In modern medicine, diseases are regarded as distinct pathological entities caused by organ dysfunction (diseases of individual organs), pathogens (infection), or cellular abnormalities (neoplasm), as registered in ICD-11^29)^ and in current medicine textbooks such as Harrison’s (2025)^30)^. In early modern medicine, illnesses were understood primarily as symptomatic conditions, such as headache or diarrhea, as enumerated in medical practice textbooks^23)^. Second, diagnostic methods evolved. Modern medicine relies on objective laboratory tests and pathological anatomy, whereas early modern diagnosis depended largely on patients’ subjective complaints, life histories, and uncertain signs such as uroscopy and pulse examination^5)^. Third, therapeutic goals shifted. Modern medicine aims to eliminate the underlying causes of disease, while early modern medicine focused mainly on alleviating severe symptoms.

In early modern Japan, physicians gradually introduced Western medicine as it evolved from traditional to modern forms. The first major step was the publication of Kaitai Shinsho (1774), which promoted the translation of Dutch anatomical texts. The second step was Siebold’s stay in Japan (1823-1829), which enhanced translation skills. Subsequently, pre-modern surgical textbooks were studied. The third step was Pompe van Meerdervoort’s medical education activities in Nagasaki (1857-1862), which facilitated the introduction of modern medicine. After this period, semi-modern textbooks were widely translated. The fourth step was Sato Susumu’s study in Germany and his introduction of modern surgery to Japan, leading to the dissemination of fully modern medical textbooks. (Table 14)

**Table 14:** Transplant of the developing modern medicine into Japan during the early three generations of directors of Juntendo

**1) Kaitai Shinsho (1774) and development of Rangaku thereafter.** => Translation of Dutch medical books. Sato Taizen studied Dutch medicine under Adachi Choshun. **2) Siebold’s stay in Japan (1823-29) and further development of Rangaku thereafter.** => Improvement and spread of Dutch translation skills. Sato Taizen studied Dutch language in Nagasaki from disciples of Siebold (1835-38). Pre-modern surgery textbook of Chelius “Handbuch der Chirurgie zum Gebrauche bei seinen Vorlesungen” (1822-23). **3) Pompe van Meerdervoort’s medical teaching in Nagasaki (1857-62)** => Introduction of the modern medicine in Japan. Sato Takanaka studied the modern medicine in Nagasaki (1860/11-62/1). Semi-modern surgery textbook of Stromeyer “Handbuch der Chirurgie. Band 1” (1844). Semi-modern medical textbook of Wunderlich “Grundriss der speciellen Pathologie und Therapie” (1858). **4) Sato Susumu’s study abroad in Germany (1869/6-75/7).** => Beginning of the modern surgery in Japan. Sato Susumu introduced the modern surgery in Japan. Modern surgery practice and teaching by Sato Susumu (1875-). Modern medicine textbook of Niemeyer “Lehrbuch der speciellen Pathologie und Therapie” (1858-61).

## Conclusion

This study analyzed the history of medical education and practice in nineteenth-century Europe and early Juntendo. It demonstrated that Western medicine underwent rapid modernization in the nineteenth century, particularly in surgery through anesthesia and disinfection, and in internal medicine through new disease concepts, objective diagnostics, and curative therapies. Early Juntendo, led by its first three directors, systematically imported and disseminated Western medicine as it evolved through three phases: the pre-modern phase (1838- 1862), the semi-modern phase (1862-1875), and the modern phase (from 1875 onward). These phases corresponded to the study trip of Sato Taizen and Sato Takanaka in Nagasaki, and of Sato Susumu in Germany. By adopting the most advanced medical knowledge of each period, the early leaders of Juntendo established a leading medical school and hospital, contributing significantly to the modernization of Japanese medicine.

## Data availability

Not applicable. No datasets were generated or analysed during the current study.

## Author contributions

TS wrote and checked the manuscript.

## Conflicts of interest statement

The author declare that there are no conflicts of interest.

## References

[1] Juntendo: Juntendo-shi Jo (“History of Juntendo, first volume”). Tokyo: Juntendo, 1980. [published in Japanese]

[2] Sakai T (ed): Nihon Igaku Kyoiku-shi (“History of medical education in Japan”). Sendai: Tohoku University Press, 2012. [published in Japanese]

[3] Sakai T: History of medical education in europe (2)—The establishment and characteristics of modern Western medicine since the 19th century. In: Sakai T, ed. History of Medical Education: Old/New and East/West. Tokyo: Hosei-daigaku Shuppankyoku, 2019: 55-140. [published in Japanese]

[4] Lindemann M: Medicine and society in early modern Europe. Second ed. Cambridge University Press, 2010.

[5] Nutton V: Renaissance medicine: a short history of European medicine in the sixteenth century. London: Routledge, 2022.

[6] National Institute of Informations: CiNii Books. Available online: https://ci.nii.ac.jp/books/ (accessed on 24 January 2026)

[7] National Diet Library: NDL digital collections. Available online: https://dl.ndl.go.jp/ (accessed on 24 January 2026)

[8] National Library of Medicine: NLM Catalog. Available online: https://www.ncbi.nlm.nih.gov/nlmcatalog?cmd=search (accessed on 24 January 2026)

[9] Bayerische Staatsbibliothek: BSB discover. Available online: https://opacplus.bsb-muenchen.de/discovery/search?vid=49BVB_BSB:VU1 (accessed on 24 January 2026)

[10] National Library of the Netherlands: KB catalogus. Available online: https://webggc.oclc.org/cbs/DB=2.37/ (accessed on 24 January 2026)

[11] Internet Archive. Available online: https://archive.org/ (accessed on 24 January 2026)

[12] Google Books. Available online: https://books.google.co.jp/advanced_book_search (accessed on 24 January 2026)

[13] Chelius MJ: Handbuch der Chirurgie zum Gebrauche bei seinen Vorlesungen. Heidelberg: Groos, 1822-1823.

[14] Stromeyer GFL: Handbuch der Chirurgie. Band 1. Freiburg im Breisgau: Herder’sche, 1844.

[15] Sato S: Geka Tsuron (“Surgery, general discussions”). Tokyo: Eirando, 1882. [published in Japanese]

[16] Sato S: Geka Kakuron (“Surgery, specific discussions”) revised, 7 vols. Tokyo: Sato Susumu, 1903-07. [published in Japanese]

[17] Wunderlich CRA: Grundriss der speciellen Pathologie und Therapie. Stuttgart: Ebner & Seubert, 1858.

[18] Niemeyer F: Lehrbuch der speciellen Pathologie und Therapie, mit besonderer Rücksicht auf Physiologie und pathologische Anatomie. Berlin: Hirschwald, 1858-1861.

[19] Sakai T: History of medical education in europe (1)—Education in the western traditional medicine before the 18th century. In: Sakai T, ed. History of Medical Education: Old/New and East/West. Tokyo: Hosei-daigaku Shuppankyoku, 2019: 5-54. [published in Japanese]

[20] Sakai T: Leonhart Fuchs and medical humanism in the history of medicine, based on an analysis of his bibliography and published books. Jap Soc Hist Med, 2025; 71: 148-176. [published in Japanese]

[21] Sakai T, Sawai T: The life and publications of Daniel Sennert (1572-1637). Jap Soc Hist Med, 2013; 59: 487-502. [published in Japanese]

[22] Sakai T, Sawai T: Boerhaave (1668-1738) and his “Institutiones medicae”. Jap Soc Hist Med, 2012; 58: 357-372. [published in Japanese]

[23] Sakai T: Genealogy of the books of Practica medicinae in Europe before the end of 18th century: from the origin to the disappearance. Jap Soc Hist Med, 2015; 61: 235-253. [published in Japanese] 26775338

[24] Sakai T: The nosology by Suvages (1706-1767). Itan, 2010; 91: 109-123. [published in Japanese]

[25] Sakai T: Natural history of the body. Tokyo: Iwanami Shoten, 2008. [published in Japanese]

[26] Sakai T, Fukushima M: Inheritance and compilation of Dioscorides’ “De materia medica” in the history of medicine: a bibliographic study of the printed books. Jap Soc Hist Med, 2023; 69: 30-48. [published in Japanese]

[27] Sakai T: European herbals in the history of medicine: a bibliographical study of printed books. Jap Soc Hist Med, 2023; 69: 363-384. [published in Japanese]

[28] Sakai T: The evolution of clinical medical books in the 19th century. Jap Soc Hist Med, 2011; 57: 19-37. [published in Japanese] 21797055

[29] WHO: ICD-11, International Classification of Diseases 11th Revision, The global standard for diagnostic health information. Available online: https://icd.who.int/en/ (Accessed on 24 January 2026)

[30] Longo DL (ed): Harrison’s principles of internal medicine. New York: McGraw Hill, 2025.

